# Supplementary material for: Proteomic Portraits Reveal Evolutionarily Conserved and Divergent Responses to Spinal Cord Injury
Source: Mol Cell Proteomics. 2021 Jun 12;20:100096. doi: 10.1016/j.mcpro.2021.100096 (PMC8260874; doi:10.1016/j.mcpro.2021.100096)
Supplement: Supplemental Table S1 [file mmc1.pdf]

Supplementary Table 1. Hyperparameter grids used for classification problems.

Classifier	Abbr.	Implementation	Hyperparameters	Values
Gaussian naive Bayes	NB	`sklearn.naive_bayes.`<br>`GaussianNB`		
K-nearest neighbours	KNN	`sklearn.neighbors.`<br>`KNeighborsClassifier`	`n_neighbors`<br>`weights`<br>`metric`	`np.arange(1, 21, 2)`<br>`['distance', 'uniform']`<br>`['euclidean', 'manhattan', 'chebyshev']`
Nearest centroid	NC	`sklearn.neighbors.`<br>`NearestCentroid`	`metric`<br>`shrink_threshold`	`['euclidean', 'manhattan', 'chebyshev']`<br>`np.arange(0, 0.05, 0.01) and`<br>`np.arange(0.05, 1, 0.05)`
Support vector machine	SVM	`sklearn.svm.SVC`	`C`<br>`kernel`<br>`gamma`<br>`coef0`	`np.logspace(-3, 2, 11)`<br>`['poly', 'rbf', 'sigmoid']`<br>`np.logspace(-2, 2, 5), 'auto', 'scale'`<br>`np.logspace(-2, 2, 9), 0`
Support vector machine, linear kernel	SVM	`sklearn.svm.LinearSVC`	`loss`<br>`penalty`<br>`dual`<br>`C`<br>`fit_intercept`	`['l1', 'l2']`<br>`['hinge', 'squared_hinge']`<br>`[True, False]`<br>`np.logspace(-10, 1, 23)`<br>`[True, False]`
Penalized logistic regression	LR	`sklearn.linear_model.`<br>`LogisticRegression`	`penalty`<br>`dual`<br>`C`<br>`fit_intercept`	`['l1', 'l2']`<br>`[True, False]`<br>`np.logspace(-10, 1, 23)`<br>`[True, False]`
Linear discriminant analysis	LDA	`sklearn.`<br>`discriminant_analysis.`<br>`LinearDiscriminantanalysis`	`solver`<br>`shrinkage`	`['svd', 'lsqr', 'eigen']`<br>`None, 'auto', np.arange(0, 1, 0.1)`
Random forests	RF	`sklearn.ensemble.`<br>`RandomForestClassifier`	`n_estimators`<br>`criterion`<br>`max_depth`<br>`max_features`<br>`min_samples_leaf`<br>`bootstrap`	`[50, 100, 500, 1000]`<br>`['gini', 'entropy']`<br>`[1, 2, 3, 4, 5, 6]`<br>`['auto', 'sqrt', 'log2']`<br>`[1, 2]`<br>`[True, False]`

Extra trees	ET	sklearn.ensemble.<br>ExtraTreesClassifier	n_estimators<br>criterion<br>max_depth<br>max_features<br>min_samples_leaf<br>bootstrap	[50, 100, 500, 1000]<br>['gini', 'entropy']<br>[1, 2, 3, 4, 5, 6]<br>['auto', 'sqrt', 'log2']<br>[1, 2]<br>[True, False]
Gradient boosting machine	GBM	sklearn.ensemble.<br>GradientBoostingClassifier	loss<br>learning_rate<br>n_estimators<br>subsample<br>max_depth<br>min_samples_leaf	['deviance', 'exponential']<br>[0.001, 0.01, 0.1, 0.25, 0.5, 1.0]<br>[50, 100, 500, 1000]<br>[0.5, 0.8, 1.0]<br>[1, 2, 3, 4, 5, 6]<br>[1, 2]
AdaBoost	ADA	sklearn.ensemble.<br>AdaBoostClassifier	n_estimators<br>learning_rate<br>algorithm	[50, 100, 500, 1000]<br>[1e-3, 1e-2, 0.01, 0.1, 0.25, 0.5, 1.0]<br>['SAMME', 'SAMME.R']
XGBoost	XGB	xgboost.sklearn.<br>XGBClassifier	max_depth<br>learning_rate<br>n_estimators<br>subsample<br>colsample_bytree<br>gamma	[1, 2, 3, 4, 5, 6]<br>[0.001, 0.01, 0.1, 0.25, 0.5, 1.0]<br>[50, 100, 500, 1000]<br>[0.5, 0.8, 1.0]<br>[0.5, 0.8, 1]<br>[0, 1, 2, 5]
