# Supplementary material for: Proteomic Portraits Reveal Evolutionarily Conserved and Divergent Responses to Spinal Cord Injury
Source: Mol Cell Proteomics. 2021 Jun 12;20:100096. doi: 10.1016/j.mcpro.2021.100096 (PMC8260874; doi:10.1016/j.mcpro.2021.100096)
Supplement: Supplemental Table S2 [file mmc2.pdf]

Supplementary Table 2. Hyperparameter grids used for regression problems.

Regressor	Abbr.	Implementation	Hyperparameters	Values
K-nearest neighbours	KNN	`sklearn.neighbors.KNeighborsClassifier`	`n_neighbors`<br>`weights`<br>`metric`	`[1, 21, 2]`<br>`['distance', 'uniform']`<br>`['euclidean', 'manhattan', 'chebyshev']`
Lasso regression	L1	`sklearn.linear_model.Lasso`	`alpha`<br>`fit_intercept`<br>`selection`	`np.logspace(-5, 4, 28)`<br>`[True, False]`<br>`['random', 'cyclic']`
Ridge regression	L2	`sklearn.linear_model.Ridge`	`alpha`<br>`fit_intercept`	`np.logspace(-5, 4, 28)`<br>`[True, False]`
ElasticNet	L1+L2	`sklearn.linear_model.ElasticNet`	`alpha`<br>`l1_ratio`<br>`fit_intercept`<br>`selection`	`np.logspace(-5, 4, 28)`<br>`np.arange(0.05, 1, 0.05)`<br>`[True, False]`<br>`['random', 'cyclic']`
Lasso fit with least-angle regression	LARS	`sklearn.linear_model.LassoLars`	`alpha`<br>`fit_intercept`	`np.logspace(-5, 4, 28)`<br>`[True, False]`
Lasso fit with least-angle regression and information criterion-based model selection	LARS	`sklearn.linear_model.LassoLarsIC`	`criterion`<br>`fit_intercept`	`['aic', 'bic']`<br>`[True, False]`
Support vector machine	SVM	`sklearn.svm.SVR`	`C`<br>`kernel`<br>`gamma`<br>`coef0`<br>`epsilon`	`np.logspace(-3, 2, 11)`<br>`['poly', 'rbf', 'sigmoid']`<br>`['auto', 'scale', np.logspace(-2, 2, 5)]`<br>`[0, np.logspace(-2, 2, 9)]`<br>`np.logspace(-2, 1, 7)`
Support vector machine, linear kernel	SVM	`sklearn.svm.LinearSVR`	`epsilon`<br>`C`<br>`fit_intercept`<br>`dual`<br>`loss`	`np.logspace(-2, 1, 7)`<br>`np.logspace(-3, 2, 11)`<br>`[True, False]`<br>`[True, False]`<br>`['epsilon_insensitive', 'squared_epsilon_insensitive']`

Random forests	RF	sklearn.ensemble.<br>RandomForestRegressor	n_estimators<br>criterion<br>max_depth<br>max_features<br>bootstrap	[50, 100, 500, 1000]<br>['mse', 'mae']<br>[1, 2, 3, 4, 5, 6]<br>['auto', 'sqrt', 'log2']<br>[True, False]
Extra trees	ET	sklearn.ensemble.<br>ExtraTreesRegressor	n_estimators<br>criterion<br>max_depth<br>max_features<br>bootstrap	[50, 100, 500, 1000]<br>['mse', 'mae']<br>[1, 2, 3, 4, 5, 6]<br>['auto', 'sqrt', 'log2']<br>[True, False]
Gradient boosting machine	GBM	sklearn.ensemble.<br>GradientBoostingRegressor	loss<br>learning_rate<br>n_estimators<br>subsample<br>max_depth	['ls', 'lad', 'huber', 'quantile']<br>[0.001, 0.01, 0.1, 0.25, 0.5, 1.0]<br>[50, 100, 500, 1000]<br>[0.5, 0.8, 1.0]<br>[1, 2, 3, 4, 5, 6]
AdaBoost	ADA	sklearn.ensemble.<br>AdaBoostRegressor	n_estimators<br>learning_rate<br>loss	[50, 100, 500, 1000]<br>[1e-3, 1e-2, 0.01, 0.1, 0.25, 0.5, 1.0]<br>['linear', 'square', 'exponential']
XGBoost	XGB	xgboost.sklearn.<br>XGBRegressor	max_depth<br>learning_rate<br>n_estimators<br>subsample<br>colsample_bytree<br>gamma	[1, 2, 3, 4, 5, 6]<br>[0.001, 0.01, 0.1, 0.25, 0.5, 1.0]<br>[50, 100, 500, 1000]<br>[0.5, 0.8, 1.0]<br>[0.8, 1]<br>[0, 1, 5]
