# Supplementary material for: Proteomic Portraits Reveal Evolutionarily Conserved and Divergent Responses to Spinal Cord Injury
Source: Mol Cell Proteomics. 2021 Jun 12;20:100096. doi: 10.1016/j.mcpro.2021.100096 (PMC8260874; doi:10.1016/j.mcpro.2021.100096)
Supplement: Supplemental Table S11 [file mmc11.pdf]

**Supplementary Table 11.** Hyperparameter grids used for regression problems.

| Regressor | Abbr. | Implementation | Hyperparameters | Values |
|---|---|---|---|---|
| K-nearest neighbours | KNN | `sklearn.neighbors.KNeighborsClassifier` | `n_neighbors`<br>`weights`<br>`metric` | `[1, 21, 2]`<br>`['distance', 'uniform']`<br>`['euclidean', 'manhattan', 'chebyshev']` |
| Lasso regression | L1 | `sklearn.linear_model.Lasso` | `alpha`<br>`fit_intercept`<br>`selection` | `np.logspace(-5, 4, 28)`<br>`[True, False]`<br>`['random', 'cyclic']` |
| Ridge regression | L2 | `sklearn.linear_model.Ridge` | `alpha`<br>`fit_intercept` | `np.logspace(-5, 4, 28)`<br>`[True, False]` |
| ElasticNet | L1+L2 | `sklearn.linear_model.ElasticNet` | `alpha`<br>`l1_ratio`<br>`fit_intercept`<br>`selection` | `np.logspace(-5, 4, 28)`<br>`np.arange(0.05, 1, 0.05)`<br>`[True, False]`<br>`['random', 'cyclic']` |
| Lasso fit with least-angle regression | LARS | `sklearn.linear_model.LassoLars` | `alpha`<br>`fit_intercept` | `np.logspace(-5, 4, 28)`<br>`[True, False]` |
| Lasso fit with least-angle regression and information criterion-based model selection | LARS | `sklearn.linear_model.LassoLarsIC` | `criterion`<br>`fit_intercept` | `['aic', 'bic']`<br>`[True, False]` |
| Support vector machine | SVM | `sklearn.svm.SVR` | `C`<br>`kernel`<br>`gamma`<br>`coef0`<br>`epsilon` | `np.logspace(-3, 2, 11)`<br>`['poly', 'rbf', 'sigmoid']`<br>`['auto', 'scale', np.logspace(-2, 2, 5)]`<br>`[0, np.logspace(-2, 2, 9)]`<br>`np.logspace(-2, 1, 7)` |
| Support vector machine, linear kernel | SVM | `sklearn.svm.LinearSVR` | `epsilon`<br>`C`<br>`fit_intercept`<br>`dual`<br>`loss` | `np.logspace(-2, 1, 7)`<br>`np.logspace(-3, 2, 11)`<br>`[True, False]`<br>`[True, False]`<br>`['epsilon_insensitive', 'squared_epsilon_insensitive']` |

| | | | | |
|---|---|---|---|---|
| Random forests | RF | `sklearn.ensemble.`<br>`RandomForestRegressor` | n_estimators<br>criterion<br>max_depth<br>max_features<br>bootstrap | [50, 100, 500, 1000]<br>['mse', 'mae']<br>[1, 2, 3, 4, 5, 6]<br>['auto', 'sqrt', 'log2']<br>[True, False] |
| Extra trees | ET | `sklearn.ensemble.`<br>`ExtraTreesRegressor` | n_estimators<br>criterion<br>max_depth<br>max_features<br>bootstrap | [50, 100, 500, 1000]<br>['mse', 'mae']<br>[1, 2, 3, 4, 5, 6]<br>['auto', 'sqrt', 'log2']<br>[True, False] |
| Gradient boosting machine | GBM | `sklearn.ensemble.`<br>`GradientBoostingRegressor` | loss<br>learning_rate<br>n_estimators<br>subsample<br>max_depth | ['ls', 'lad', 'huber', 'quantile']<br>[0.001, 0.01, 0.1, 0.25, 0.5, 1.0]<br>[50, 100, 500, 1000]<br>[0.5, 0.8, 1.0]<br>[1, 2, 3, 4, 5, 6] |
| AdaBoost | ADA | `sklearn.ensemble.`<br>`AdaBoostRegressor` | n_estimators<br>learning_rate<br>loss | [50, 100, 500, 1000]<br>[1e-3, 1e-2, 0.01, 0.1, 0.25, 0.5, 1.0]<br>['linear', 'square', 'exponential'] |
| XGBoost | XGB | `xgboost.sklearn.`<br>`XGBRegressor` | max_depth<br>learning_rate<br>n_estimators<br>subsample<br>colsample_bytree<br>gamma | [1, 2, 3, 4, 5, 6]<br>[0.001, 0.01, 0.1, 0.25, 0.5, 1.0]<br>[50, 100, 500, 1000]<br>[0.5, 0.8, 1.0]<br>[0.8, 1]<br>[0, 1, 5] |
