# Supplementary material for: Proteomic Portraits Reveal Evolutionarily Conserved and Divergent Responses to Spinal Cord Injury
Source: Mol Cell Proteomics. 2021 Jun 12;20:100096. doi: 10.1016/j.mcpro.2021.100096 (PMC8260874; doi:10.1016/j.mcpro.2021.100096)
Supplement: Supplemental Figures S1–S12 [file mmc12.docx]

Supplemental Figures

**
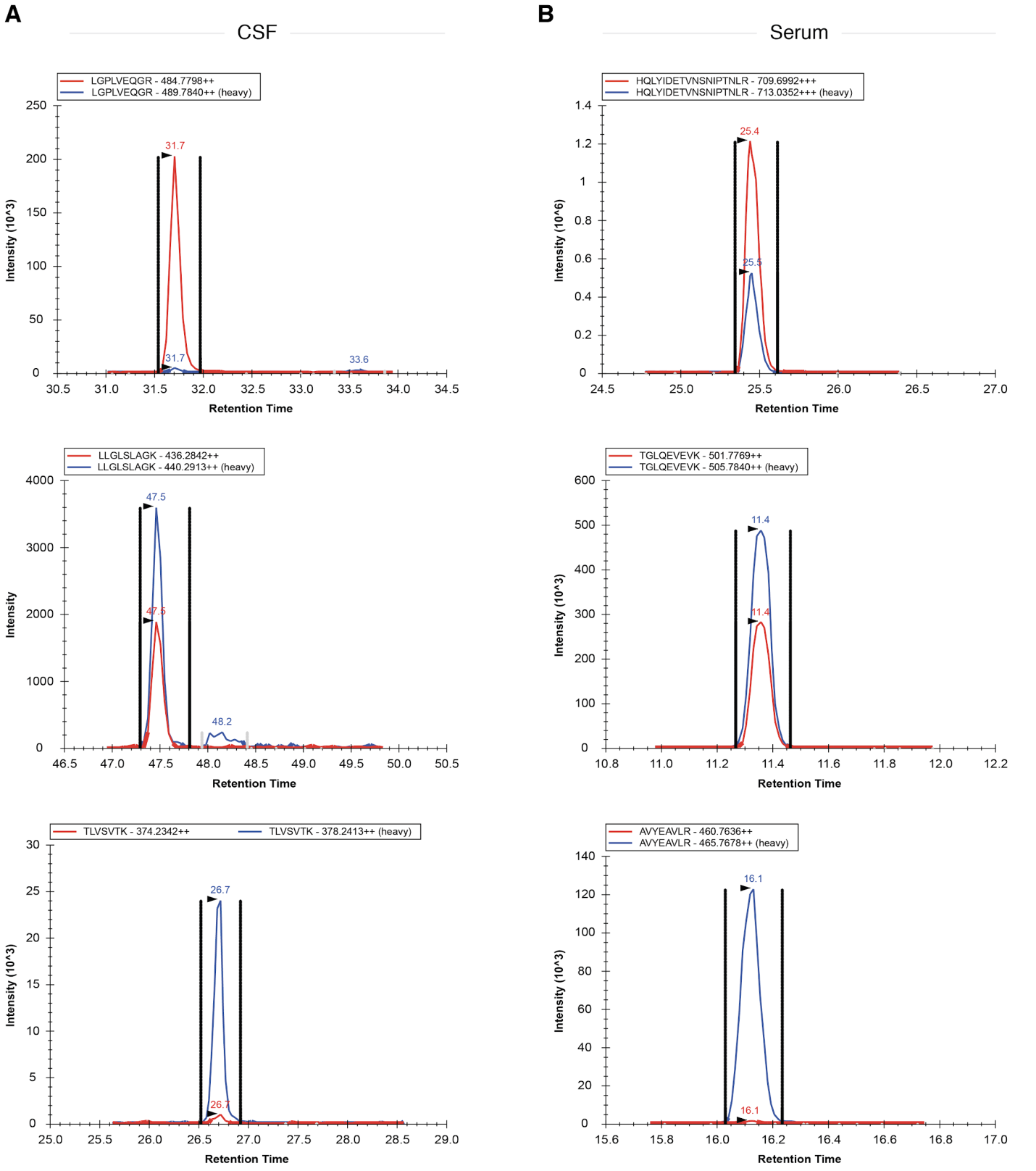
**

**Supplemental Figure 1. Example MRM and PRM data.**(**A**) Example PRM data in the CSF for peptides with high (top), moderate (middle), and low (bottom) ratios to the respective heavy synthetic peptides.
(**B**) As in (**A**), but for the MRM data in the serum.

**
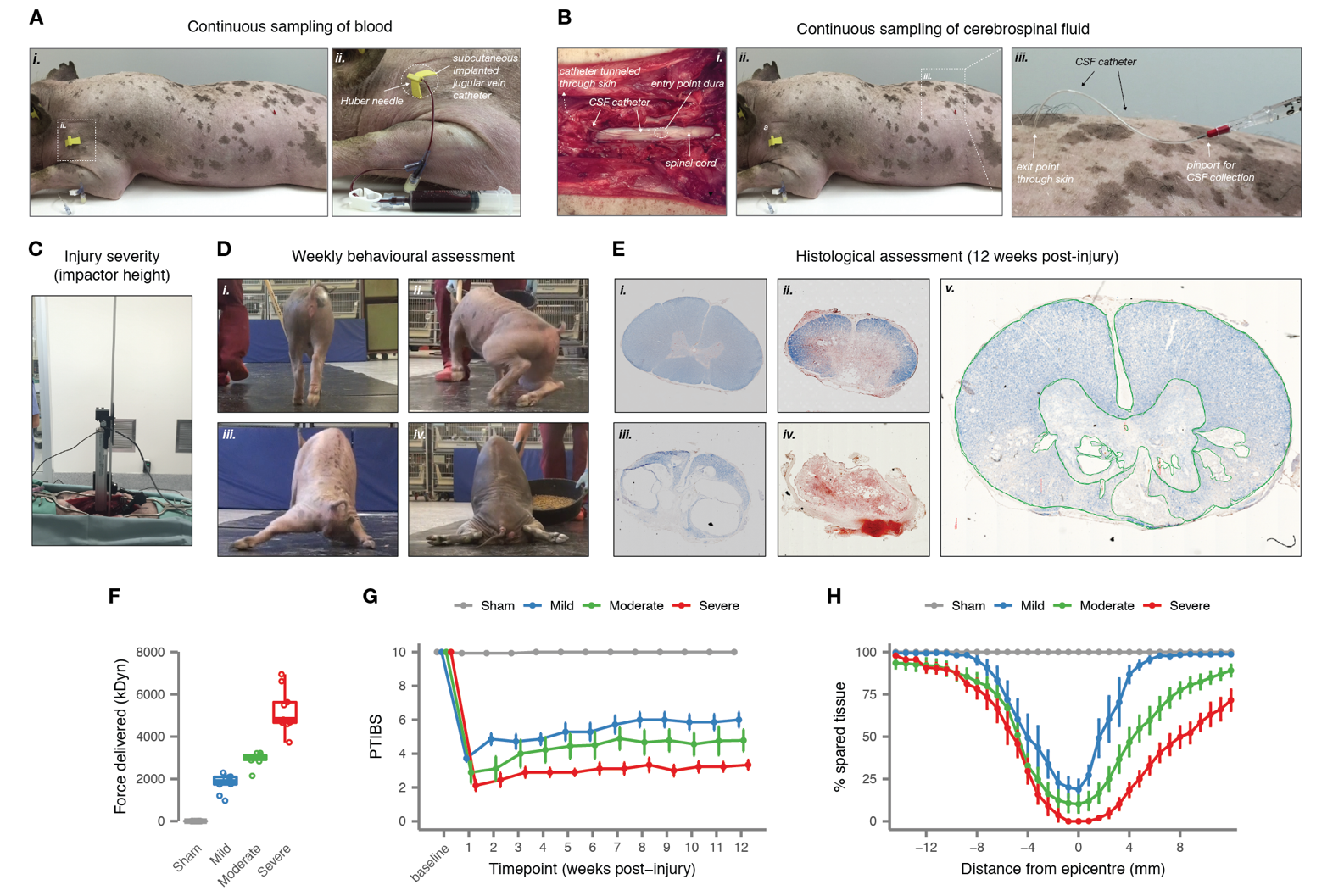
**

**Supplemental Figure 2. Experimental study of SCI in a large animal model, the Yucatan pig.**(**A-B**) Overview of surgical and experimental setup for continuous collection of CSF (**A**) and serum (**B**) samples. 
(**C-E**) Overview of key experimental outcomes. 
(**C**) Baseline injury severity was quantified by impactor height (mild, 10 cm; moderate, 20 cm; severe, 40 cm).
(**D**) Neurological recovery was quantified by weekly behavioral assessments using the Porcine Thoracic Injury Behavioral Scale (PTIBS) for hindlimb function. Representative images from PTIBS assessments are shown for sham (i), mild (ii), moderate (iii), and severe (iv) injuries. 
(**E**) Tissue sparing was quantified by histology at 12 weeks post-injury, on the basis of cross-sections of the spinal cord from 13.6 mm rostral to 13.6 mm caudal to the lesion site, in increments of 0.8 mm. Slides were manually traced to determine the extent of spared tissue (v).
(**F**) Maximum force delivered, in kDyn, to animals in each of four treatment groups.
(**G**) Mean PTIBS scores for animals in each of four treatment groups over the first twelve weeks post-injury. Bars show standard error. 
(**H**) Mean proportion of spared tissue in sections taken from 13.6 mm rostral to 13.6 mm caudal to the lesion, relative to the entire area of the spinal cord on histological section. Bars show standard error.

**
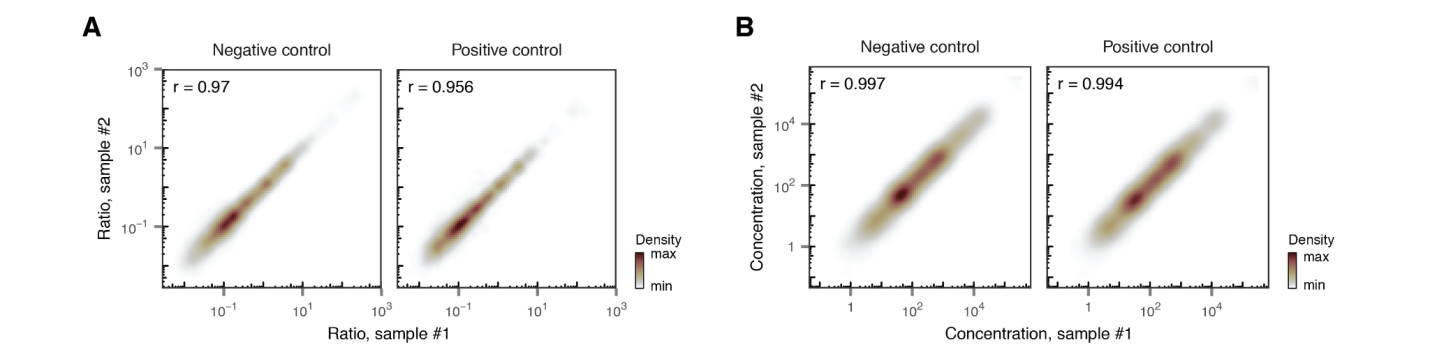
Supplemental Figure 3. Reproducibility of CSF and serum proteome measurements.**(**A**) Two-dimensional density plot showing the similarity of replicated measurements between pairs of pooled control samples in the CSF. Inset text shows the correlation coefficient.
(**B**) As in (**A**), but for pooled control samples in the serum.


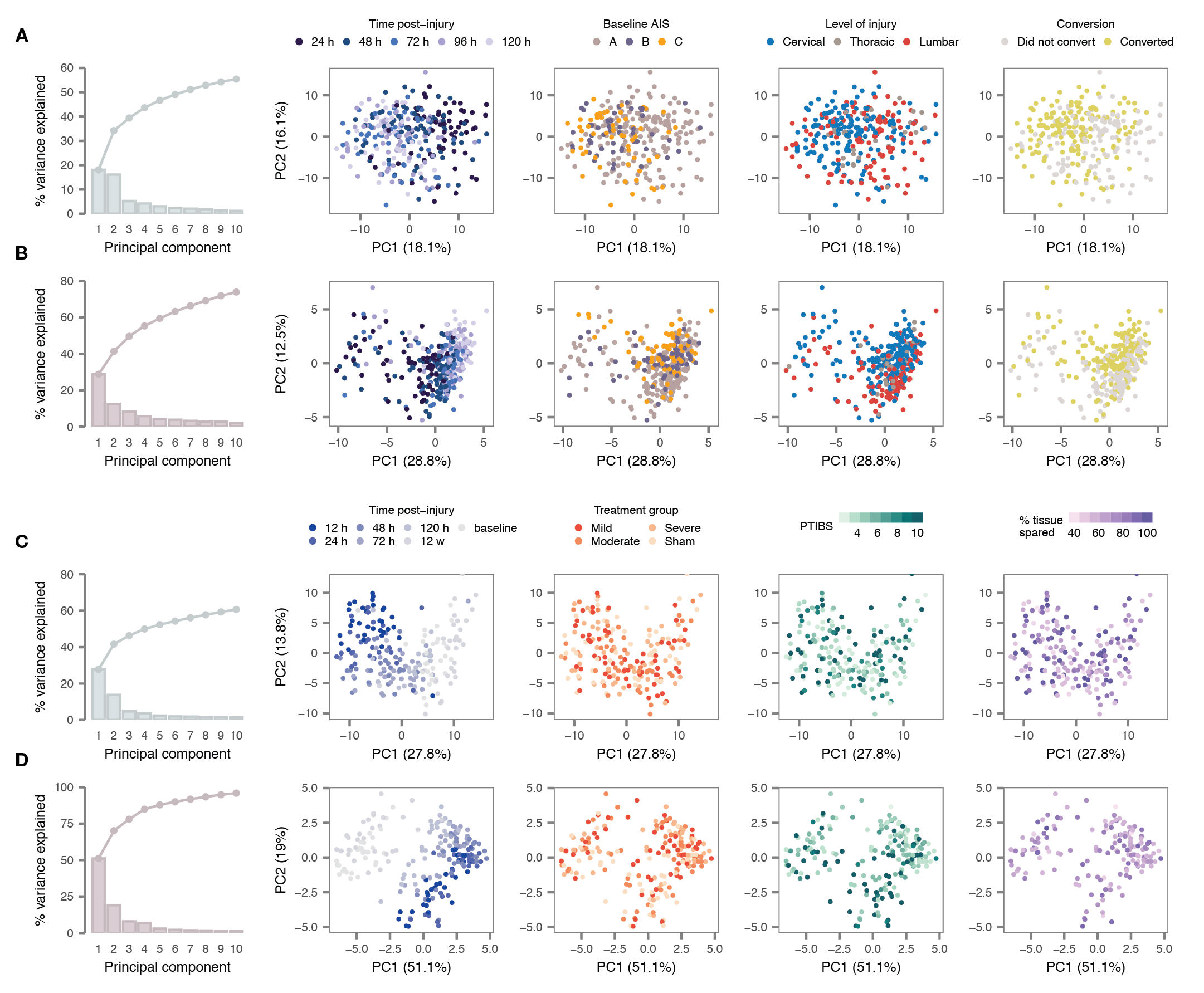


**Supplemental Figure 4. Principal component analysis of CSF and serum proteomes in human and pig.**(**A**) Principal component analysis of CSF proteomics data from the human cohort. Left, percent of variance explained by principal components 1 to 10 (bars) or cumulatively by the first 1 to 10 principal components (line). Right, biplot of the first two principal components (PC1 and PC2) with samples coloured by time post-injury, baseline AIS grade, level of injury, or AIS conversion.
(**B**) As in (**A**) but for the serum proteomics data from the human cohort.
(**C**) Principal component analysis of CSF proteomics data from the pig cohort. Left, percent of variance explained by principal components 1 to 10 (bars) or cumulatively by the first 1 to 10 principal components (line). Right, biplot of the first two principal components (PC1 and PC2) with samples coloured by time post-injury, treatment group (baseline injury severity), PTIBS score at 12 weeks post-injury, and percentage of tissue spared as assessed by histology at 12 weeks post-injury.
(**D**) As in (**C**) but for the serum proteomics data from the pig cohort.

**
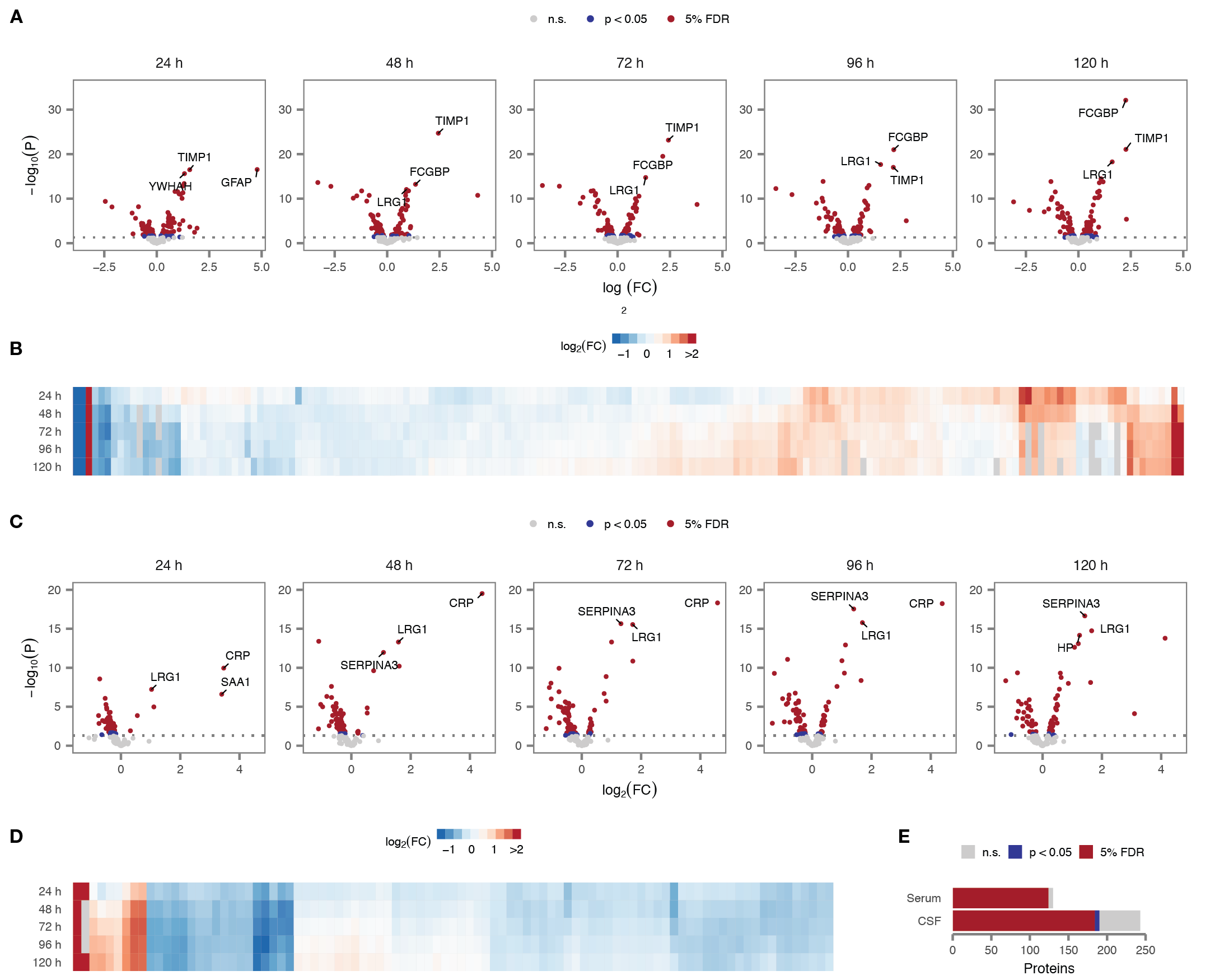
**

**Supplemental Figure 5. Alterations in CSF and serum protein abundance in acute SCI.**(**A**) Volcano plots of differential protein abundance between 24 h and 120 h post-injury in CSF samples from patients with acute SCI compared to uninjured controls. 
(**B**) Time courses of differential protein abundance (log-fold change, relative to uninjured controls) over the first five days post-injury for 171 CSF proteins differentially expressed between SCI and control samples within at least one timepoint. 
(**C**) As in (**A**) but showing differential abundance of serum proteins compared to uninjured controls. 
(**D**) As in (**B**) but showing time courses of 92 serum proteins differentially expressed between SCI and control samples within at least one timepoint. 
(**E**) Numbers of CSF and serum proteins that display a statistically significant association with time post-injury.


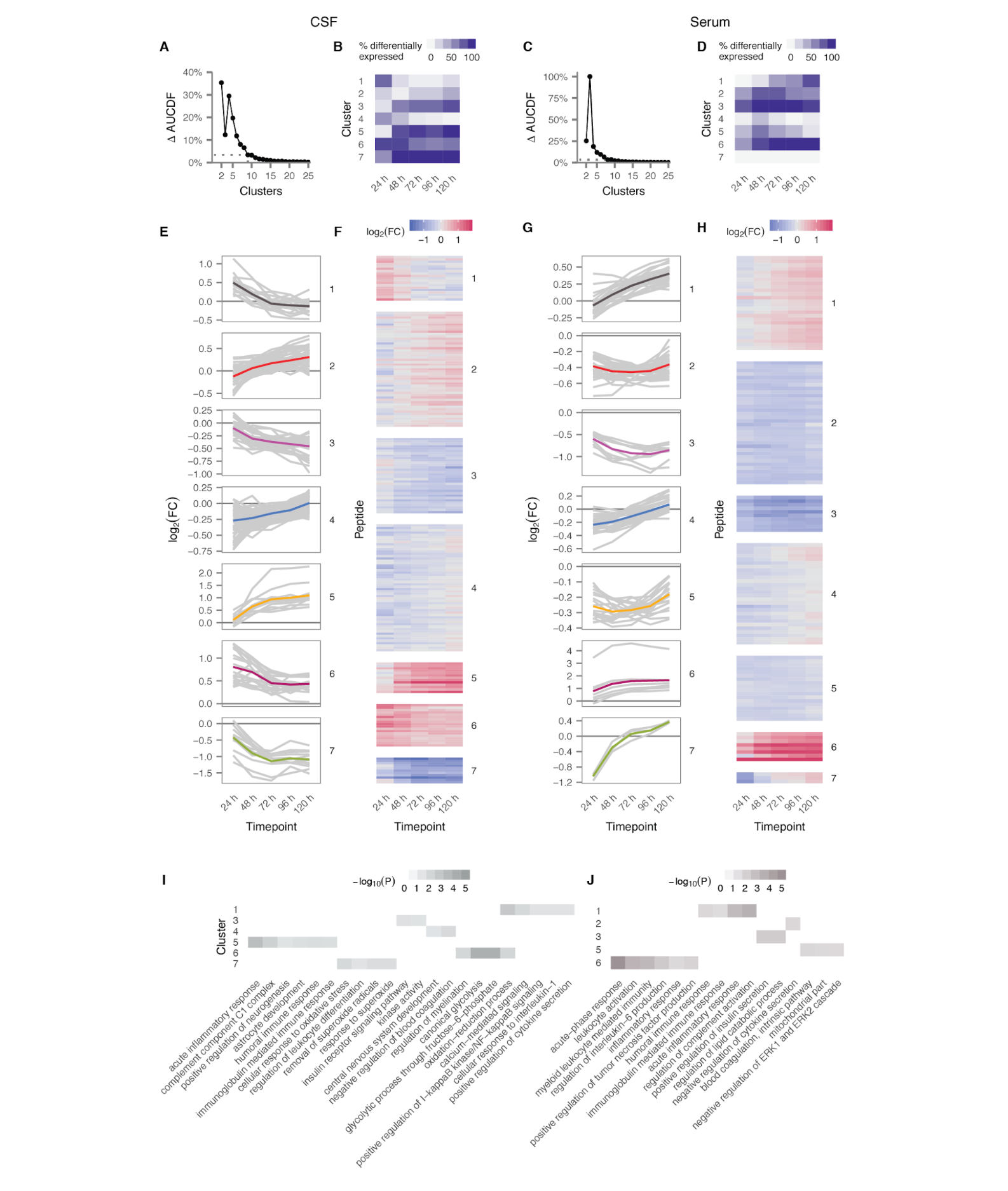


**Supplemental Figure 6. Modules of temporally co-regulated CSF and serum proteins in acute SCI.**(**A**) Relative change in the area under the cumulative distribution function comparing consensus clustering solutions of *k* and *k – 1* clusters of the CSF proteome. The optimal number of clusters was determined for each biofluid as the value of *k* at which there was no appreciable increase in the AUCDF. 
(**B**) Proportion of peptides from each cluster found to be differentially expressed between acute SCI patients and uninjured controls at each timepoint. 
(**C-D**) As in (**A-B**) but for the serum proteome.
(**E**) Time courses of differential protein abundance (log_2_-fold change, relative to uninjured controls) over the first five days post-injury for peptides in each of the seven CSF protein modules, grey lines, and the mean time course for the entire module, colored line. 
(**F**) Time courses of differential protein abundance, as in (**C**), shown as a heatmap. 
(**G-H**) As in (**E-F**) but for the serum proteome.
(**I-J**) GO terms enriched in each of each of the seven CSF (**I**) and serum (**J**) protein modules.


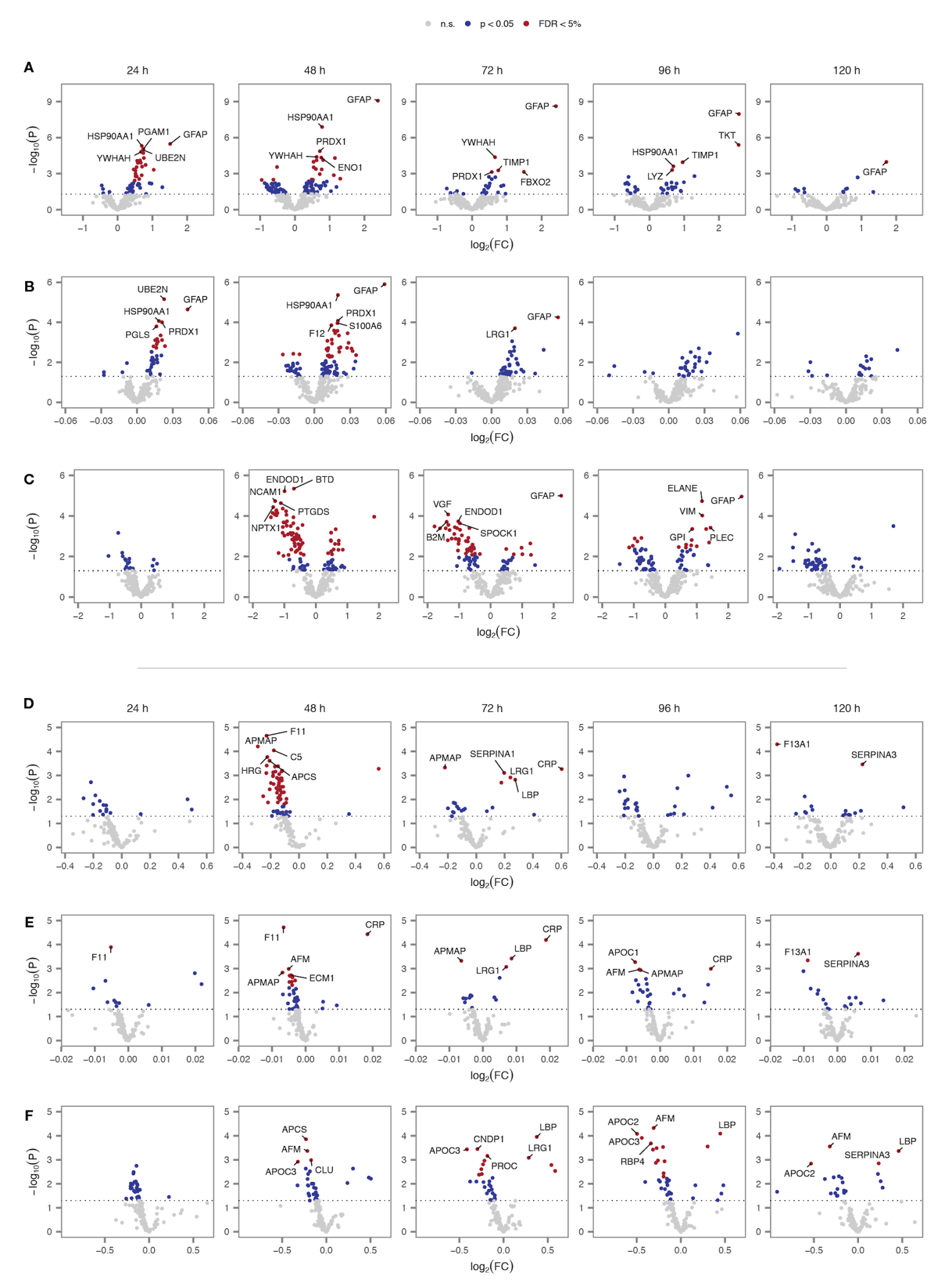


**Supplemental Figure 7. Univariate analysis of injury severity and neurological recovery in human serum and CSF.**
(**A**) Volcano plots of differential protein abundance as a function of injury severity, as quantified by the baseline AIS grade, in CSF samples between 24 h and 120 h post-injury.
(**B**) Volcano plots of differential protein abundance as a function of neurological recovery at six months post-injury, as quantified by the change in TMS relative to baseline, in CSF samples between 24 h and 120 h post-injury.
(**C**) Volcano plots of differential protein abundance as a function of neurological recovery at six months post-injury, as quantified by improvement in the AIS grade relative to baseline, in CSF samples between 24 h and 120 h post-injury.
(**D-F**) As in (**A-C**), respectively, but for serum samples.


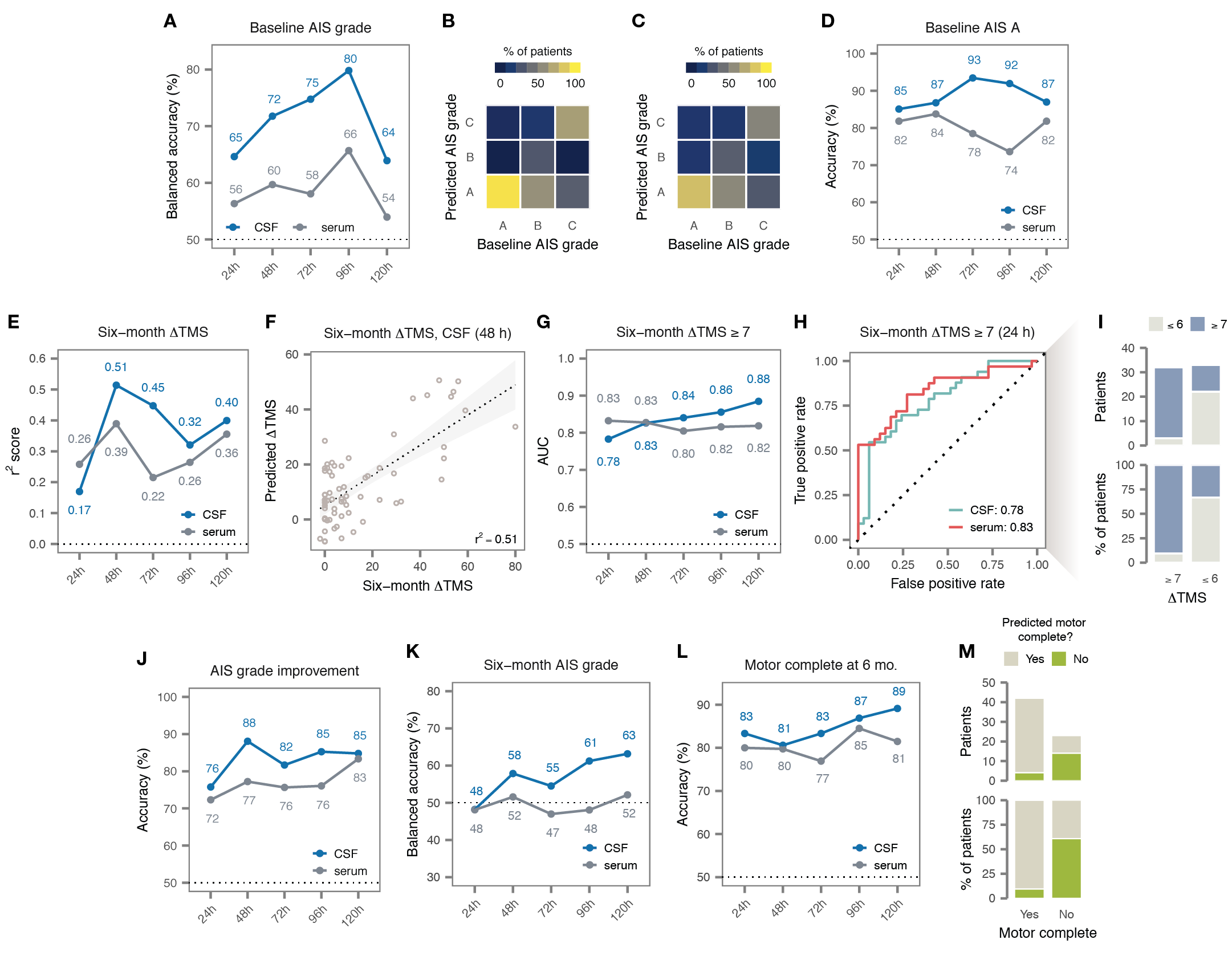


**Supplemental Figure 8. Additional multivariate analysis of SCI severity and recovery.**(**A**) Cross-validation balanced accuracy of multivariate diagnostic models trained to stratify patients by baseline AIS grade, at timepoints between 24 and 120 h post-injury.
(**B**) Confusion matrix of the best CSF diagnostic model of baseline AIS grade at 24 h post-injury.
(**C**) Confusion matrix of the best serum diagnostic model of baseline AIS grade at 24 h post-injury.
(**D**) Cross-validation accuracy of multivariate diagnostic models trained to discern patients with a baseline AIS grade of A, at timepoints between 24 and 120 h post-injury.
(**E**) Cross-validation coefficient of determination (r^2^ score) of multivariate prognostic models trained to predict the change in TMS at six months post-injury, relative to baseline, at timepoints between 24 and 120 h post-injury.
(**F**) Predictions made by the best CSF prognostic model of six-month change in TMS at 48 h post-injury. 
(**G**) Cross-validation AUC of multivariate prognostic models trained to predict a change in TMS of seven or more points at six months post-injury, at timepoints between 24 and 120 h post-injury.
(**H**) ROC curves of the best CSF and serum prognostic models of six-month change in TMS of seven or more points at 24 h post-injury. 
(**I**) Predictions made by the best serum prognostic model at 24 h post-injury. 
(**J**) Cross-validation accuracy of multivariate prognostic models trained to predict improvement in AIS grade at six months post-injury, relative to baseline, at timepoints between 24 and 120 h post-injury.
(**K**) Cross-validation balanced accuracy of multivariate prognostic models trained to predict AIS grade at six months, at timepoints between 24 and 120 h post-injury.
(**L**) Cross-validation accuracy of multivariate prognostic models trained to predict motor complete vs. incomplete injury at six months, at timepoints between 24 and 120 h post-injury.
(**M**) Predictions made by the best serum prognostic model of motor complete vs. incomplete injury at six months, at 24 h post-injury.


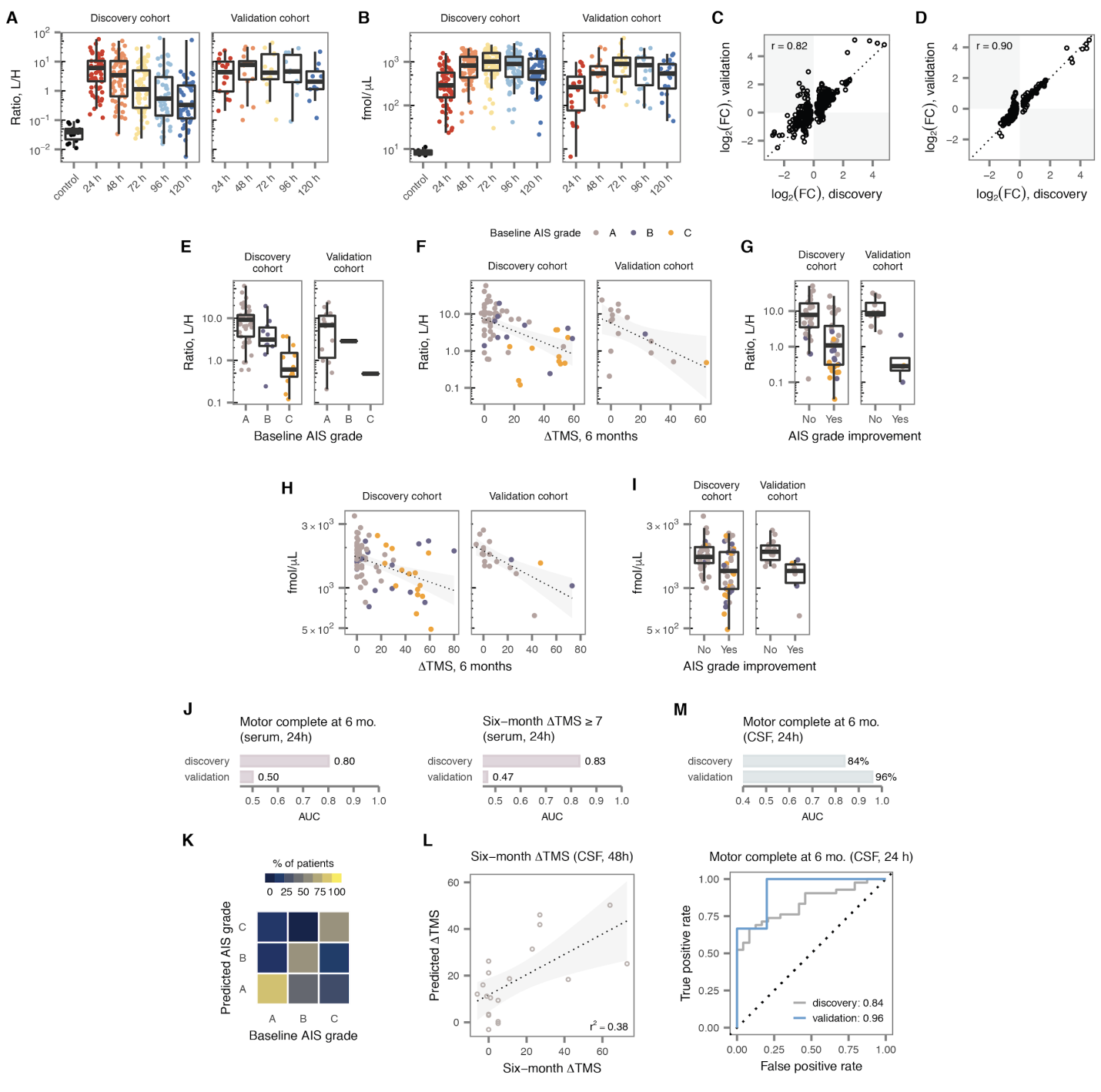


**Supplemental Figure 9. Replication of univariate associations and multivariate models in an independent validation cohort.**(**A**) Time course of CSF abundance for an exemplary protein with replicated alterations between acute SCI patients and uninjured controls, GFAP. 
(**B**) Time course of serum abundance for an exemplary protein with replicated alterations between acute SCI patients and uninjured controls, CRP. 
(**C**) Correlation between log_2_-fold changes between the discovery and validation cohorts for proteins with a statistically significant alteration in the discovery cohort, regardless of replication status in the validation cohort.
(**D**) As in (**C**), but for serum proteins.
(**E-I**) Examples of proteins with univariate associations to severity or recovery replicated in the validation cohort.
(**E**) Abundance of GFAP in CSF samples at 24 h, stratified by baseline AIS grade.
(**F**) Abundance of GFAP in CSF samples at 24 h, stratified by change in TMS at six months post-injury.
(**G**) Abundance of GFAP in CSF samples at 48 h, stratified by improvement in AIS grade at six months post-injury.
(**H**) Abundance of LRG1 in serum samples at 72 h, stratified by change in TMS at six months post-injury. 
(**I**) Abundance of LRG1 in serum samples at 72 h, stratified by improvement in AIS grade at six months post-injury.
(**J**) Performance of two preregistered serum multivariate models in the validation cohort.
(**K**) Confusion matrix of the best CSF diagnostic model of baseline AIS grade at 48 h post-injury (as shown in Fig. 4B) in the validation cohort.
(**L**) Performance of the best CSF prognostic model of six-month change in TMS at 48 h post-injury in the validation cohort.
(**M**) Performance, top, and ROC curve, bottom, of the best CSF prognostic model of motor complete vs. incomplete injury at six months at 24 h post-injury.


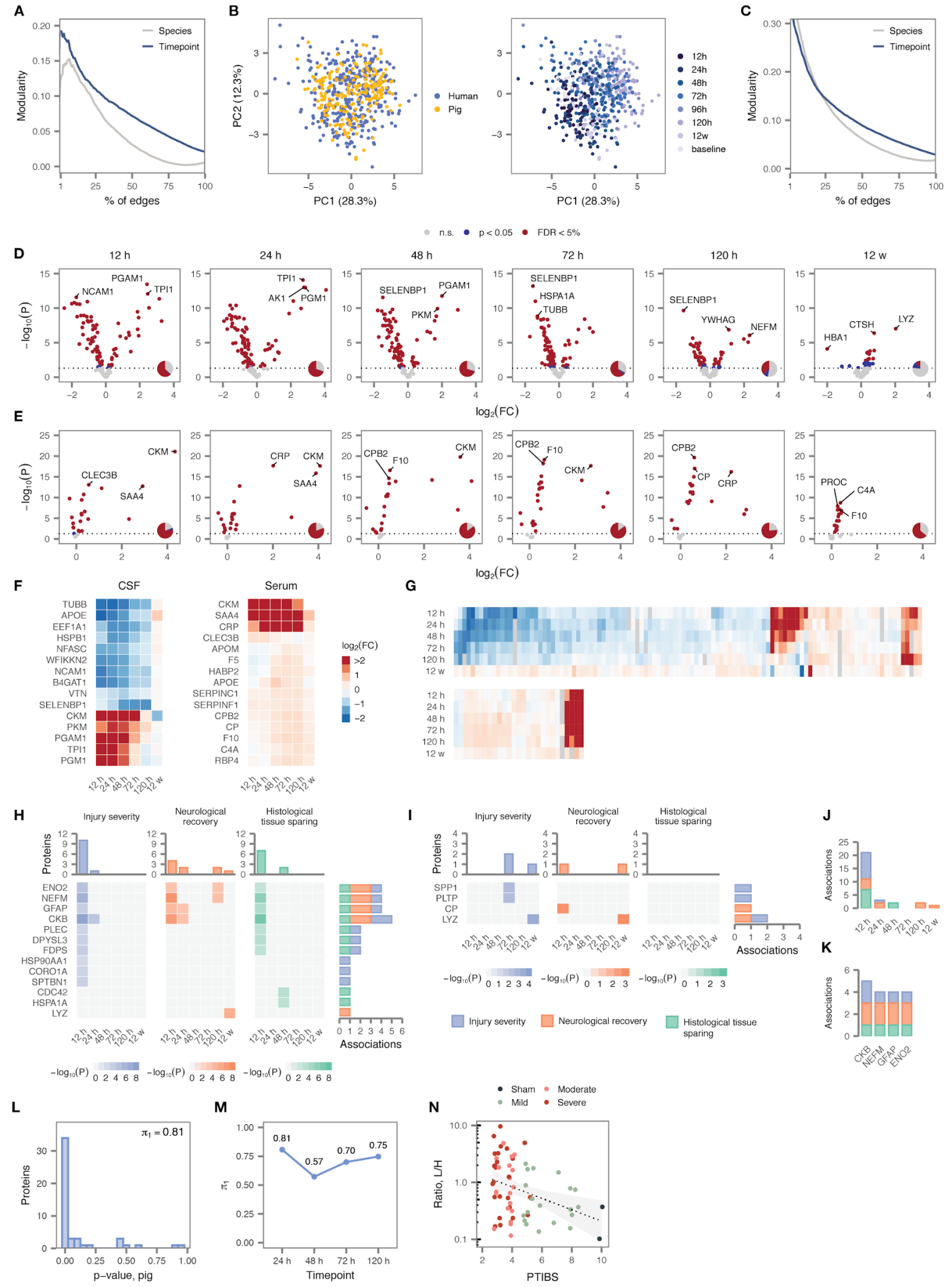


**Supplemental Figure 10. Univariate analysis of acute SCI, injury severity, and neurological recovery in pig serum and CSF.**(**A**) Modularity analysis of the combined human and pig CSF proteomes, with samples grouped by species or time post-injury. Modularity is shown as a function of the number of edges between samples used to construct the network, as a percentage of the total number of possible edges.
(**B**) Principal component analysis of human and pig serum proteomes, with samples colored by species, left, or time post-injury, right.
(**C**) As in (**A**) but for the combined serum proteomes. 
(**D**) Volcano plots of differential protein abundance between 24 h and 120 h post-injury in CSF samples from injured pigs, compared to samples drawn from the same pigs at baseline (15 min prior to injury).
(**E**) As in (**D**) but for serum protein abundance.
(**F**) Time courses of differential protein abundance (log_2_-fold change, relative to baseline) over twelve weeks post-injury for fifteen of the most profoundly altered CSF proteins, left, and serum proteins, right.
(**G**) Time courses of differential protein abundance (log_2_-fold change, relative to uninjured controls) over twelve weeks post-injury for 135 CSF proteins, top, and 26 serum proteins, bottom, differentially expressed between SCI and control samples within at least one timepoint. 
(**H**) Statistical significance of associations between CSF protein abundance and three experimental variables over twelve weeks post-injury for 13 proteins with at least one significant association. Grey squares indicate associations that were not significant after correction for multiple hypothesis testing.
(**I**) As in (**H**) but for four serum proteins with at least one significant association. 
(**J-K**) Number of statistically significant associations between CSF protein abundance and three experimental variables outcomes per timepoint (**J**) and for the four CSF proteins with the most recurrent associations (**K**).
(**L**) Distribution of p-values for proteins differentially expressed in the CSF of acute SCI patients vs. uninjured controls, in comparisons of pig CSF samples drawn at 24 h post-injury and at baseline. 
(**M**) Estimated proportion of true associations, π_1_, for all human-pig comparisons between 24 h and 120 h.
(**N**) Abundance of GFAP in pig CSF samples at 24 h, stratified by PTIBS score.


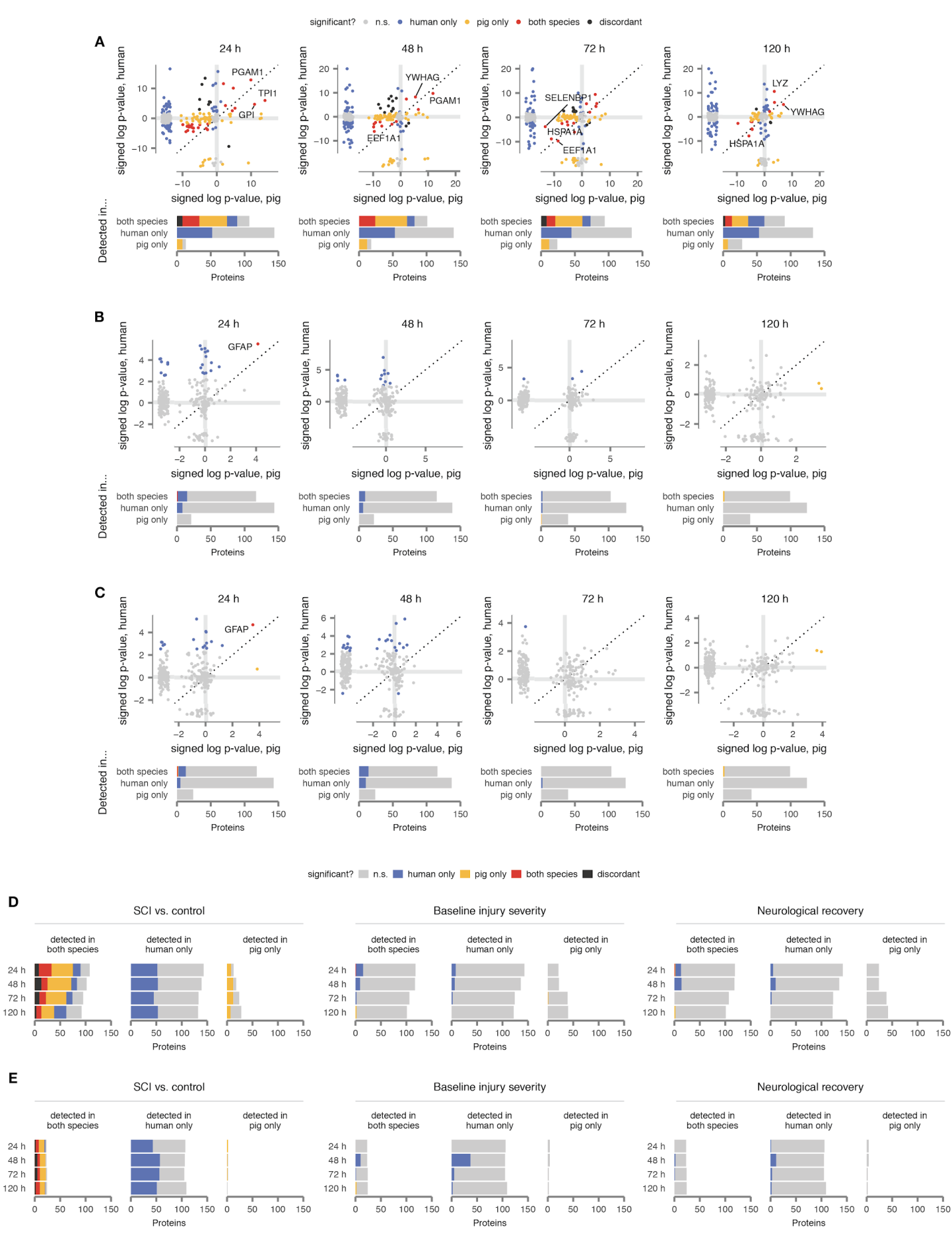


**Supplemental Figure 11. Overlap of differentially abundant proteins between human and pig.**(**A**) Top, signed –log_10_ p-values for differential protein abundance between samples from individuals with acute SCI and uninjured controls at four matching timepoints in human and pig. Marginal plots show signed –log_10_ p-values for proteins quantified in human (pig) only. Bottom, number of proteins with statistically significant differential abundance in both species, human only, pig only, or neither, among proteins quantified in both species, human only, or pig only.
(**B**) As in (**A**) but for proteins associated with injury severity. 
(**C**) As in (**A**) but for proteins associated with neurological recovery. 
(**D**) Summary of significant univariate associations involving CSF proteins across species, stratified by (i) clinical or experimental outcome, (ii) time post-injury, (iii) detection in one or both species, and (iv) statistical significance in neither, one, or both species. 
(**E**) As in (**D**) but for the serum proteome.


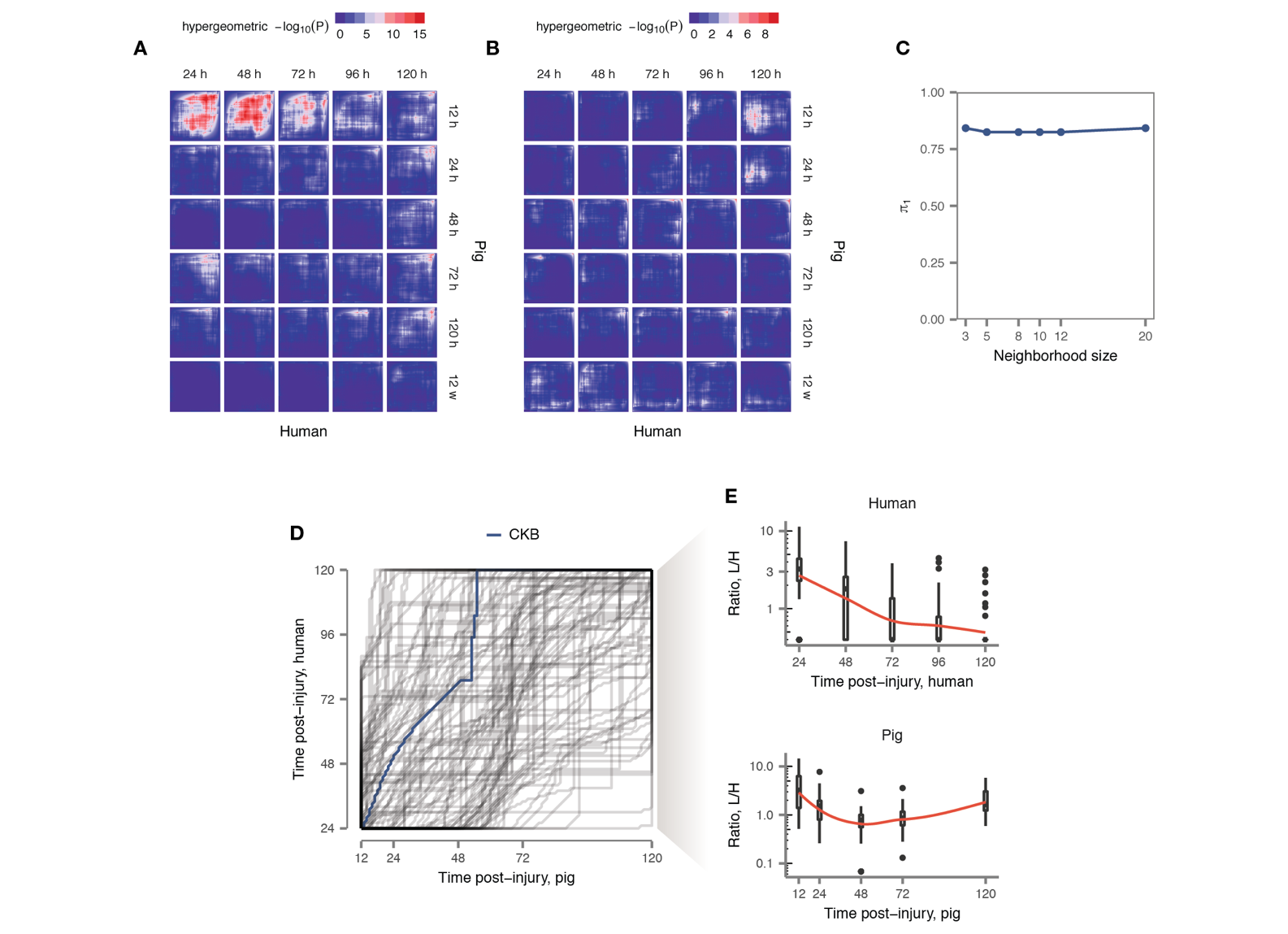


**Supplemental Figure 12. Cross-species conservation of the proteomic response to SCI.**(**A**) Rank-rank hypergeometric overlap of proteins associated with injury severity at all pairs of timepoints post-injury.
(**B**) Rank-rank hypergeometric overlap of proteins associated with neurological recovery at all pairs of timepoints post-injury.
(**C**) Proportion of true associations, π_1_, estimated from neighborhood analysis of conserved co-expression p-value distributions with the neighborhood size varied between 3 and 20 neighbors. 
(**D**) Optimal alignments of individual proteins between the human and pig CSF proteomes over the first five days post-injury by dynamic time warping. CKB is shown in blue. 
(**E**) Abundance of CKB in human and pig CSF over the first five days post-injury. Red lines show local polynomial (loess) regression.
